# Propensity score analysis for health care disparities: a deweighting approach

**DOI:** 10.1186/s12874-024-02230-5

**Published:** 2024-05-03

**Authors:** Byeong Yeob Choi

**Affiliations:** Department of Population Health Sciences, UT Health San Antonio, 7703 Floyd Curl Drive, Mail Code 7933, San Antonio, 78229 TX USA

**Keywords:** Absolute standardized mean difference, Balancing weight, Deweighting, Health care disparity, Health status, Propensity score, Socioeconomic status

## Abstract

**Background:**

Propensity score weighting is a useful tool to make causal or unconfounded comparisons between groups. According to the definition by the Institute of Medicine (IOM), estimates of health care disparities should be adjusted for health-status factors but not for socioeconomic status (SES) variables. There have been attempts to use propensity score weighting to generate estimates that are concordant with IOM’s definition. However, the existing propensity score methods do not preserve SES distributions in minority and majority groups unless SES variables are independent of health status variables.

**Methods:**

The present study introduces a deweighting method that uses two types of propensity scores. One is a function of all covariates of health status and SES variables and is used to weight study subjects to adjust for them. The other is a function of only the SES variables and is used to deweight the subjects to preserve the original SES distributions.

**Results:**

The procedure of deweighting is illustrated using a dataset from a right heart catheterization (RHC) study, where it was used to examine whether there was a disparity between black and white patients in receiving RHC. The empirical example provided promising evidence that the deweighting method successfully preserved the marginal SES distributions for both racial groups but balanced the conditional distributions of health status given SES.

**Conclusions:**

Deweighting is a promising tool for implementing the IOM-definition of health care disparities. The method is expected to be broadly applied to quantitative research on health care disparities.

**Supplementary Information:**

The online version contains supplementary material available at 10.1186/s12874-024-02230-5.

## Introduction

Evaluating racial disparities needs specific statistical approaches to quantify and find solutions to reduce them. For example, Jackson and VanderWeele [1] investigated how disparities in wages would change if disparities in education were removed while preserving the association of childhood SES with race. This approach allows us to estimate how well removing disparities in education, but not childhood SES, reduces disparities in adulthood wages. Ben-Michale et al. [2] evaluated differences between black and white patients in common outcomes after emergency general surgery (EGS) treatment using an administrative database of hospital claims. They used linear weighting estimators that re-weight white patients to have a similar distribution of the adjustment set to black patients. They found that adjusting for hospitals in addition to patient characteristics substantially reduced the disparity estimate resulting from adjusting for basic demographics (age and sex). This finding implied that hospital-specific factors are important sources of racial disparities in EGS, and thus, interventions targeted at hospital quality may be critical.

In this study, we utilize the definition of the Institute of Medicine (IOM) to propose new disparity estimators. The Institute of Medicine (IOM) defines a health care disparity as the difference in the quality of health care provided to members of racial or ethnic minorities that is not due to their health status, clinical needs, or treatment preferences [3]. To implement the IOM definition, a group of researchers proposed a specific statistical approach that adjusts for health-status factors but not for nonhealth variables, i.e., socioeconomic status (SES) variables, such as education, income, and geographic region [4–6]. The rationale of this approach is that the IOM definition of disparities should include all racial differences in use mediated through SES-related factors. However, we acknowledge that this approach, adjusting for only health-status variables, is one of the ways to implement the IOM definition. Considering such a limitation and the complexity of disentangling the effects of race/ethnicity from SES factors, we have compared the proposed approach with those adjusting for all covariates and investigated how these methods give different results in a real data example.

Several researchers devised statistical models based on PS models to address the needs required by the IOM definition of racial disparities. Cook et al. [5] defined distributional properties necessary for a disparity method to be concordant with the IOM definition of racial disparities in health care use. Specifically, they compared a counterfactual white population with black health status and white SES with the factual black population. This white distribution is counterfactual because it has black health status but white SES. One of their disparity methods to construct the hypothetical white distribution is the health-status PS method. In racial disparity studies, minority race is the “treatment” of interest for the PS. The health-status PS uses only the health-status variables to predict the probability of minority race. Motivated by a disparity study of Li et al. [7], Choi et al. [8] extended the health-status PS method by adopting the balancing weights approach [9] to address the broader range of disparity measures. However, the fundamental limitation of the health-status PS method is that SES variables are not preserved unless they are independent of health status.

In this study, we propose weights that can realize IOM-concordant joint distributions of health status and SES. The key idea is that we weight the groups with the PSs using all covariates but deweight them with the PSs based on only SES. In this way, we directly undo the undesired weighting of the SES variables. Considering this feature of our approach, we call it a deweighting approach. Importantly, we formally define the target estimands for health disparities and corresponding consistent estimators based on the proposed weights.

The deweighting approach is illustrated in a study of right heart catheterization (RHC) [10] to investigate whether there was a disparity between black and white patients, admitted to the intensive care unit, in receiving RHC. Based on unadjusted proportions, black patients were 1.86% less likely to receive RHC than white patients. However, this unadjusted difference does not account for differences in health status or clinical needs, which do not contribute to disparities. In the RHC study, white patients tended to have lower blood pressure than black patients, and this could contribute to apparent disparities for black patients in receiving RHC. Because the disparities due to clinical needs such as low blood pressure are allowable, these factors need to be adjusted for. Traditional multivariable methods adjust for all available covariates, which also removes differences due to non-clinical needs. Thus, the controlled differences will better represent racial disparities if the SES variables are preserved in both racial groups so that the SES variables can mediate the relationship between race and RHC treatment. This motivates the development of our dweighting approach.

## Methods

### Notation

We introduce notation to describe our deweighting approach. For unit $i=1,\dots , N$, let ${Z}_{i}$ be the binary variable for minority race/ethnicity and ${Y}_{i}$ be the observed health care outcome. In our numerical study, we assessed whether there was a disparity in receiving RHC at the time of admission to the intensive care unit between black and white patients: ${Y}_{i}=1$ if the patient received RHC, and ${Y}_{i}=0$ otherwise. Therefore, in our analysis, ${Z}_{i}=1$ and ${Z}_{i}=0$ indicate black and white patients, respectively. For causal inference, $Y\left(1\right)$ and $Y\left(0\right)$ are defined as the potential outcomes corresponding to treatment levels 1 and 0. The observed outcome is then $Y=ZY\left(1\right)+\left(1-Z\right)Y\left(0\right)$ under the consistency assumption.

Let ${X}_{i}$ be a row vector of $J+K$ covariates consisting of SES and health-status variables: ${X}_{i}=({X}_{i}^{s}, {X}_{i}^{h})$, where ${X}_{i}^{s}=\left({X}_{i1}^{s}, \dots , {X}_{iJ}^{s}\right)$ is a row vector of $J$ SES variables, and ${X}_{i}^{h}=\left({X}_{i,J+1}^{h}, \dots , {X}_{i,J+K}^{h}\right)$ is a row vector of $K$ health-status variables. Let $f\left(x\right)$ and ${f}_{z}\left(x\right)$ denote the densities of $X$ in the whole population and subpopulation of $Z=z$, and $f\left({x}^{s}\right)$ and ${f}_{z}\left({x}^{s}\right)$ denote those of ${X}^{s}$ in the whole population and subpopulation of $Z=z$, respectively, where $z\in \{0, 1\}$. We also define $f\left({x}^{h}\mid {x}^{s}\right)$ and ${f}_{z}\left({{x}^{h}\mid x}^{s}\right)$ to be the conditional densities of ${X}^{h}$ given ${X}^{s}={x}^{s}$ for the whole population and subpopulation of $Z=z$.

### Propensity scores

We define the full PS as a function of all available covariates:

$$e\left(x\right)=\text{Pr}(Z=1\mid X=x).$$

In health disparities research, we are interested in comparing health care outcomes between the minority group (coded as $$Z=1)$$ with the majority group (coded as $$Z=0$$). Therefore, in our study, the PS represents the probability of being in the minority group conditional on the covariates. The standard causal inference approach adopts the full PS to balance all covariates between comparison groups. However, balancing all covariates does not comply with the IOM’s definition because the original differences in the SES variables cannot be preserved. To overcome this issue from using the full PS, Cook et al. [5] proposed using the health-status PS, which is a function of only the health-status variables:

$$e\left({x}^{h}\right)=\text{Pr}\left(Z=1\vert{X}^{h}={x}^{h}\right).$$

Choi et al. [8] extended the approach of Cook et al. [5] to study various estimands of health care disparities. Weighting based on the health-status PS is optimal if the health status and SES variables are independent of each other, but it generally alters the SES variables, as demonstrated by Choi et al. [8]. To address this issue, Choi et al. [8] introduced a data-adaptive method to find the balancing weights that minimize the alternations of the SES variables while preventing severe imbalance in the health-status variables. This data-adaptive method might be effective in overcoming the inherent limitation of the health-status PS method but is still an indirect way to preserve SES variables.

Rather than using the health-status PSs, the proposed deweighting approach weights the subjects with the full PSs but deweights them using the PSs based on only the SES variables. Because the SES variables are involved in the weights, the proposed approach directly preserves the SES variables, while adjusting for the health-status variables. We will refer to the PS using only the SES variables as the SES-PS, defined as:

$$e\left({x}^{s}\right)=\text{Pr}\left(Z=1\vert{X}^{s}={x}^{s}\right).$$

### Balancing weights

To use the PSs to study health disparities, we adopt the balancing weights approach [9]. The balancing weights based on the full PSs are defined as

1 $$\begin{array}{c}{\omega }_{1}^{B}\left(x\right)=\frac{g\left(x\right)}{e\left(x\right)}, and\ {\omega }_{0}^{B}\left(x\right)=\frac{g\left(x\right)}{1-e\left(x\right)}\end{array}$$

where $g\left(x\right)$ is a selection function that determines the target population of interest. If we weight the treated group by ${\omega }_{1}^{B}\left(x\right)$ and the control group by ${\omega }_{0}^{B}\left(x\right)$, then ${f}_{1}\left(x\right)$ and ${f}_{0}\left(x\right)$ will be balanced toward $t\left(x\right)=g\left(x\right)f\left(x\right)/E\left\{g\left(x\right)\right\}$. Typically, $g\left(x\right)$ is a function of $e\left(x\right)$. Therefore, it is worthwhile to look at the target population when $g\left(x\right)$ is equal to $e\left(x\right)$ or $1-e\left(x\right)$:

2 $$\begin{array}{c}{f}_{1}\left(x\right)\propto e\left(x\right)f\left(x\right), and\ {f}_{0}\left(x\right)\propto \left\{1-e\left(x\right)\right\}f\left(x\right)\end{array}$$

Based on Eq. (2), we can demonstrate that ${f}_{1}\left(x\right){\omega }_{1}^{B}\left(x\right)\propto g\left(x\right)f\left(x\right)$ and ${f}_{0}\left(x\right){\omega }_{0}^{B}\left(x\right)\propto g\left(x\right)f\left(x\right)$, and therefore ${\omega }_{1}^{B}\left(x\right)$ and ${\omega }_{0}^{B}\left(x\right)$ are called the balancing weights.

The specification of $g\left(x\right)$ determines the target populations and estimands, as shown in Table 1 of Li et al. [9]. For example, when $g\left(x\right)=1$, the estimand is the average treatment effect (ATE), and the weights are the inverse probability weights (IPWs), $\left\{\omega_1^B\left(x\right),\omega_0^B\left(x\right)\right\}=\left\{1/e\left(x\right),1/(1-e\left(x\right))\right\}$. When $g\left(x\right)=e\left(x\right)$, the estimand is the average treatment effect on the treated (ATT), and the weights are the standardized mortality ratio weights (SMRWs), $\left\{\omega_1^B\left(x\right),\omega_0^B\left(x\right)\right\}=\left\{1,e(x)/(1-e\left(x\right))\right\}$. Li et al. [9] introduced overlap weights to identify the average treatment effect in the overlap population (ATO), where $g\left(x\right)=e\left(x\right)\left\{1-e\left(x\right)\right\}$, and the weights are $\left\{\omega_1^B\left(x\right),\omega_0^B\left(x\right)\right\}=\left\{1-e\left(x\right),e(x)\right\}$. The overlap weights are useful to address extreme PSs [11].

**Table 1:** Baseline characteristics of the overall, black non-RHC, black RHC, white non-RHC, and white RHC patients

	Overall	Black non-RHC	Black RHC	Whie non-RHC	White RHC
N	5380	585	335	2753	1707
Age (mean (SD))	61.99 (16.46)	57.42 (17.79)	54.88 (16.87)	63.45 (16.64)	62.60 (14.90)
Sex = Male (%)	2994 (55.7)	290 (49.6)	165 (49.3)	1505 (54.7)	1034 (60.6)
Cancer (%)					
Metastatic	362 (6.7)	49 (8.4)	18 (5.4)	198 (7.2)	97 (5.7)
No cancer	4105 (76.3)	466 (79.7)	276 (82.4)	2029 (73.7)	1334 (78.1)
Localized	913 (17.0)	70 (12.0)	41 (12.2)	526 (19.1)	276 (16.2)
Disease category (%)					
ARF	2354 (43.8)	255 (43.6)	121 (36.1)	1244 (45.2)	734 (43.0)
CHF	422 (7.8)	42 (7.2)	37 (11.0)	193 (7.0)	150 (8.8)
MOSF	1504 (28.0)	146 (25.0)	140 (41.8)	557 (20.2)	661 (38.7)
Other	1100 (20.4)	142 (24.3)	37 (11.0)	759 (27.6)	162 (9.5)
Number of comorbidities (mean (SD))	1.51 (1.16)	1.42 (1.17)	1.35 (1.07)	1.56 (1.17)	1.50 (1.14)
Duke activity status index (mean (SD))	20.48 (5.31)	20.45 (5.81)	20.82 (5.25)	20.30 (5.39)	20.73 (5.00)
APACHE score (mean (SD))	54.59 (19.88)	52.56 (19.99)	62.82 (20.94)	50.48 (18.30)	60.31 (20.21)
Glasgow coma score (mean (SD))	21.03 (30.48)	26.06 (32.47)	21.63 (30.65)	21.50 (31.26)	18.44 (28.14)
Mean blood pressure (mean (SD))	78.56 (38.16)	93.76 (42.08)	70.64 (39.42)	83.10 (38.00)	67.59 (33.28)
WBC (mean (SD))	15.77 (11.98)	15.87 (13.25)	16.54 (14.36)	15.27 (11.09)	16.40 (12.36)
Heart rate (mean (SD))	114.98 (41.52)	113.88 (42.04)	118.64 (46.20)	112.58 (40.93)	118.52 (41.06)
Respiratory rate (mean (SD))	28.05 (14.12)	29.86 (15.16)	26.63 (15.29)	28.74 (13.74)	26.61 (13.98)
Temperature (mean (SD))	37.61 (1.78)	37.53 (1.92)	37.34 (2.01)	37.65 (1.71)	37.64 (1.79)
PaO2/FiO2 ratio (mean (SD))	221.31 (114.15)	261.19 (121.04)	214.19 (118.05)	234.86 (114.28)	187.20 (101.30)
Albumin (mean (SD))	3.09 (0.79)	3.13 (0.63)	2.88 (0.76)	3.17 (0.68)	2.99 (0.97)
Hematocrit (mean (SD))	31.95 (8.38)	31.06 (8.72)	29.52 (7.71)	33.19 (8.75)	30.75 (7.40)
Bilirubin (mean (SD))	2.11 (4.37)	2.04 (4.54)	2.25 (3.95)	1.82 (3.81)	2.58 (5.12)
Creatinine (mean (SD))	2.13 (2.05)	2.49 (2.82)	2.87 (2.51)	1.81 (1.80)	2.38 (1.92)
Sodium (mean (SD))	136.81 (7.63)	138.21 (7.69)	136.42 (7.69)	136.81 (7.59)	136.42 (7.60)
Potassium (mean (SD))	4.07 (1.02)	4.11 (1.14)	4.13 (1.09)	4.07 (1.00)	4.03 (0.99)
PaCO2 (mean (SD))	38.80 (13.15)	38.30 (13.24)	36.77 (10.93)	40.40 (14.34)	36.78 (11.00)
PH (mean (SD))	7.39 (0.11)	7.39 (0.10)	7.37 (0.11)	7.39 (0.11)	7.38 (0.11)
Weight (mean (SD))	68.25 (29.05)	65.43 (30.83)	75.01 (27.10)	65.46 (29.17)	72.40 (27.89)
‘Do not resuscitate’ status on day 1 = Yes (%)	626 (11.6)	63 (10.8)	16 (4.8)	414 (15.0)	133 (7.8)
Insurance (%)					
Government	2338 (43.5)	361 (61.7)	186 (55.5)	1196 (43.4)	595 (34.9)
Private	2752 (51.2)	174 (29.7)	111 (33.1)	1440 (52.3)	1027 (60.2)
Uninsured	290 (5.4)	50 (8.5)	38 (11.3)	117 (4.2)	85 (5.0)
High school education or more (%)	1527 (28.4)	102 (17.4)	69 (20.6)	779 (28.3)	577 (33.8)
Income >=$25k (%)	1258 (23.4)	63 (10.8)	44 (13.1)	649 (23.6)	502 (29.4)

Let $\tau \left(x\right)=E\left[Y\mid Z=1,X=x\right]-E\left[Y\mid Z=0,X=x\right]$ denote the conditional average controlled difference given $X=x$ [12]. Based on Hirano et al. [13] and Li et al. [9], we can express the target estimand as the weighted average of $\tau \left(x\right)$ over the target population, $g\left(x\right)f\left(x\right)$:

3 $$\begin{array}{c}{\tau }_{g}=\frac{\int \tau \left(x\right)g\left(x\right)f\left(x\right)dx}{\int g\left(x\right)f\left(x\right)dx}\end{array}$$

In causal inference, ${\tau }_{g}$ is called the weighted average treatment effect (WATE). The necessary conditions to identify the WATE from the observed data include the assumptions of unconfoundedness and positivity [14]. For descriptive comparisons, we will call it the weighted average controlled difference (WACD). Because race/ethnicity is not manipulable, the estimand more related to health care disparity is the WACD. However, we will adopt the terminologies used for the WATE. For example, we will use the terms ‘ATE’ and ‘ATT’ to represent the measures of health care disparity in the combined (minority plus majority) and minority groups, respectively.

Following Theorem 1 of Li et al., the consistent estimator for ${\tau }_{g}$ is

$${\widehat{\tau }}_{g}=\frac{\sum _{i}{\omega }_{1}^{B}\left({X}_{i}\right){Z}_{i}{Y}_{i}}{\sum _{i}{\omega }_{1}^{B}\left({X}_{i}\right){Z}_{i}}-\frac{\sum _{i}{\omega }_{0}^{B}\left({X}_{i}\right){(1-Z}_{i}){Y}_{i}}{\sum _{i}{\omega }_{0}^{B}\left({X}_{i}\right)\left(1-{Z}_{i}\right)}.$$

Our proposed approach is based on the balancing weights in Eq. (1) but needs additional balancing weights based on the SES-PSs:

$${\omega }_{1}^{B}\left({x}^{s}\right)=\frac{g\left({x}^{s}\right)}{e\left({x}^{s}\right)}, \text{a}\text{n}\text{d}\ {\omega }_{0}^{B}\left({x}^{s}\right)=\frac{g\left({x}^{s}\right)}{1-e\left({x}^{s}\right)}.$$

The key contribution of this study is to demonstrate that the following weights $\omega_1\left(x\right)=\frac{\omega_1^B\left(x\right)}{\omega_1^B\left(x^s\right)}$ and $\omega_0\left(x\right)=\frac{\omega_0^B\left(x\right)}{\omega_0^B\left(x^s\right)}$, can create joint distributions aligned with the IOM’s definition. Before we discuss these proposed weights, we will define the estimands for health care disparities.

### Estimands for health care disparities

It is important to note that the WATE or WACD in (3) is obtained by averaging $\tau \left(x\right)$ over the common target joint distribution of health status and SES. Accordingly, the estimand in (3) may not be appropriate as a measure of health care disparities because the SES variables are altered. Because the IOM definition requires SES to be preserved but health status to be balanced between groups, it would be desirable to balance health status conditional on SES. Therefore, our target population for each group is represented by the target conditional health-status distribution given SES times its own marginal SES distribution.

Let ${f}_{g}\left({x}^{h}\mid {x}^{s}\right)$ denote a target conditional distribution of health status given SES. We will show that ${f}_{g}\left({x}^{h}\mid {x}^{s}\right) = f \left({x}^{h}\vert{x}^{s}\right) g \left(x\right)/g\left({x}^{s}\right)$, where $g\left(x\right)$ is a selection function used to define the WATE in (3). The selection function $g\left({x}^{s}\right)$ is a function of $e({x}^s)$ but with the same form as $g\left(x\right)$. A simple example arises when $g\left(x\right)=g\left({x}^{s}\right)=1$; the target conditional distribution of health status is $f\left({x}^{h}\vert{x}^{s}\right)$, which is for the combined population. In this case, the target conditional distribution is the combined distribution of health status from minority and majority subjects, and thus, we will refer to the corresponding estimand as the ATE measure of health disparity. When $g\left(x\right)=e\left(x\right)$ and $g\left({x}^{s}\right)=e\left({x}^{s}\right)$, the target conditional distribution of health status is ${f}_{1}\left({x}^{h}\vert{x}^{s}\right)$, which is for the minority population. Because the target conditional distribution is for minority health status, we will refer to the corresponding estimand as the ATT measure of health disparity. From the fact that a joint distribution is a conditional distribution of health status given SES times a marginal distribution of SES, the target joint distribution for the group $Z=z$ can be written as ${t}_{z}\left(x\right)= {f}_{g}\left({x}^{h}\mid {x}^{s}\right){f}_{z}\left({x}^{s}\right)/E\left[{f}_{g}\left({x}^{h}\mid {x}^{s}\right){f}_{z}\left({x}^{s}\right)\right]$, where $E\left[{f}_{g}\left({x}^{h}\mid {x}^{s}\right){f}_{z}\left({x}^{s}\right)\right]$ are normalizing constants for $z=\text{0,1}$. These joint distributions have the common conditional distribution of health status ${f}_{g}\left({x}^{h}\mid {x}^{s}\right)$ but their own original marginal distributions ${f}_{1}\left({x}^{s}\right)$ and ${f}_{0}\left({x}^{s}\right)$ for minority and majority groups, respectively.

Now we define the estimand for disparity as ${{\tau }_{g}={\mu }_{g1}-\mu }_{g0}$, where ${\mu }_{g1}$ and ${\mu }_{g0}$ are:

4 $$\begin{array}{c}{\mu }_{g1}=\frac{\int E\left[Y\mid Z=1,X=x\right]{f}_{g}\left({x}^{h}\mid {x}^{s}\right){f}_{1}\left({x}^{s}\right)dx}{\int {f}_{g}\left({x}^{h}\mid {x}^{s}\right){f}_{1}\left({x}^{s}\right)dx}\end{array}$$

and

5 $$\begin{array}{c}{\mu }_{g0}=\frac{\int E\left[Y\mid Z=0,X=x\right]{f}_{g}\left({x}^{h}\mid {x}^{s}\right){f}_{0}\left({x}^{s}\right)dx}{\int {f}_{g}\left({x}^{h}\mid {x}^{s}\right){f}_{0}\left({x}^{s}\right)dx}.\end{array}$$

Note that ${\mu }_{g1}$ and ${\mu }_{g0}$ are defined by averaging the conditional expectation of $Y$ over the same target conditional distribution of health status but different marginal distributions of SES. Our hypothesis of interest is that this ${\tau }_{g}$ is equal to 0 or not.

In the next section, we will discuss important examples of the proposed weights to demonstrate how ${\mu }_{g1}$ and ${\mu }_{g0}$ can be estimated for the ATE and ATT measures of health disparities, aligned with IOM’s definition.

### Proposed weights

We illustrate our approach with two important examples, the ATE and ATT measures of health disparity. Suppose that we used the IPWs, $\{1/e\left(x\right),1/(1-e\left(x\right))$}, to estimate the ATE. Because the full PS includes SES variables, they could be altered by the weighting. To alleviate the impacts of the IPWs on the SES variables, we deweight the subjects based on the SES-PSs. That is, we multiply the SES-PSs to the minority group and the ‘1 minus SES-PSs’ to the majority group. As a result, we have the following weights:

6 $$\begin{array}{c}{\omega }_{1}\left(x\right)=\frac{e\left({x}^{s}\right)}{e\left(x\right)}, and\ {\omega }_{0}\left(x\right)=\frac{1-e\left({x}^{s}\right)}{1-e\left(x\right)}.\end{array}$$

First, we demonstrate that the joint distribution resulting from weighting the minority group with ${\omega }_{1}\left(x\right)$ is equal to the conditional distribution of health status given SES in the combined group times the minority distribution of SES:

$${f}_{1}\left(x\right){\omega }_{1}\left(x\right)\propto f\left(x\right)e\left({x}^{s}\right)=f\left({x}^{h}\vert{x}^{s}\right)f\left({x}^{s}\right)e\left({x}^{s}\right)\propto f\left({x}^{h}\vert{x}^{s}\right){f}_{1}\left({x}^{s}\right).$$

This resultant joint distribution, $f\left({x}^{h}\vert{x}^{s}\right){f}_{1}\left({x}^{s}\right)$, is counterfactual because the conditional health-status distribution is from the combined population, but the marginal SES distribution is from the minority group.

In the same way, we can show that the joint distribution resulting from weighting the majority group with ${\omega }_{0}\left(x\right)$ is equal to the conditional distribution of health status given SES in the combined group times the majority distribution of SES:

$${f}_{0}\left(x\right){\omega }_{0}\left(x\right)\propto f\left(x\right)\left\{1-e\left({x}^{s}\right)\right\}=f\left({x}^{h}\vert{x}^{s}\right)f\left({x}^{s}\right)\left\{1-e\left({x}^{s}\right)\right\}\propto f\left({x}^{h}\vert{x}^{s}\right){f}_{0}\left({x}^{s}\right).$$

Therefore, the proposed weights in Eq. (6) generate hypothetical distributions aligned with the IOM’s definition because the marginal SES distributions for both groups are kept but the target conditional distribution of health status coincides with $f\left({x}^{h}\vert{x}^{s}\right)$. Therefore, the original SES differences between the groups can contribute to the ATE estimate of health disparity, while adjusting for health status.

Now we discuss the racial disparity in the minority group, the ATT measure of health disparity. Suppose that we used the SMRWs, $\left\{1,e(x)/(1-e\left(x\right))\right\}$, to estimate the ATT. In this case, we do not deweight the minority group because all the weights are 1, which does not alter the SES variables. Because the majority subjects are weighted by $e\left(x\right)/(1-e\left(x\right))$, we multiply the majority group by the inverse of $\frac{e\left(x^s\right)}{\left\{1-e\left(x^s\right)\right\}}$ to deweight them. As a result, we have the following weights:

7 $$\begin{array}{c}{\omega }_{1}\left(x\right)=1, and\ {\omega }_{0}\left(x\right)=\frac{e\left(x\right)}{1-e\left(x\right)}{\left\{\frac{e\left({x}^{s}\right)}{1-e\left({x}^{s}\right)}\right\}}^{-1}.\end{array}$$

We can show that the joint distribution resulting from weighting the majority group with ${\omega }_{0}\left(x\right)$ in Eq. (7) is equal to the conditional distribution of health status given SES in the minority group times the majority distribution of SES:

$${f}_{0}\left(x\right){\omega }_{0}\left(x\right)\propto {f}_{1}\left(x\right){\left\{\frac{e\left({x}^{s}\right)}{1-e\left({x}^{s}\right)}\right\}}^{-1}={f}_{1}\left({x}^{h}\vert{x}^{s}\right){f}_{1}\left({x}^{s}\right)\frac{1-e\left({x}^{s}\right)}{e\left({x}^{s}\right)}\propto {f}_{1}\left({x}^{h}\vert{x}^{s}\right){f}_{0}\left({x}^{s}\right).$$

This resulting joint distribution ${f}_{1}\left({x}^{h}\vert{x}^{s}\right){f}_{0}\left({x}^{s}\right)$ is clearly counterfactual because the conditional health-status is for the minority, but the marginal SES is for the majority.

### Generalization

We can generalize the weights for the ATE and ATT as follows:

8 $$\begin{array}{c}{\omega }_{1}\left(x\right)=\frac{{\omega }_{1}^{B}\left(x\right)}{{\omega }_{1}^{B}\left({x}^{s}\right)}, and\ {\omega }_{0}\left(x\right)=\frac{{\omega }_{0}^{B}\left(x\right)}{{\omega }_{0}^{B}\left({x}^{s}\right)}.\end{array}$$

For the ATE, $\left\{\omega_1^B\left(x\right),\omega_0^B\left(x\right)\right\}=\{1/e\left(x\right),1/(1-e\left(x\right))\}$ and $\left\{{\omega}_1^B\left({x}^s\right),{\omega }_0^B\left({x}^s\right)\right\}=\{1/e\left({x}^s\right),1/(1-e\left({x}^s\right))$}. For the ATT, $\left\{\omega_1^B\left(x\right),\omega_0^B\left(x\right)\right\}=\{1,e(x)/(1-e\left(x\right))\}$ and $\left\{\omega_1^B\left(x^s\right),\omega_0^B\left(x^s\right)\right\}=\{1,e(x^s)/(1-e\left(x^s\right))\}$.

The weighted minority distribution by ${\omega }_{1}\left(x\right)$ is

$${f}_{1}\left(x\right){\omega }_{1}\left(x\right)={f}_{1}\left(x\right)\frac{g\left(x\right)}{e\left(x\right)}\frac{e\left({x}^{s}\right)}{g\left({x}^{s}\right)}\propto f\left(x\right)e\left({x}^{s}\right)\frac{g\left(x\right)}{g\left({x}^{s}\right)}\propto \left\{f\left({x}^{h}\vert{x}^{s}\right)\frac{g\left(x\right)}{g\left({x}^{s}\right)}\right\}{f}_{1}\left({x}^{s}\right).$$

In the same way, we can show that the weighted majority distribution by ${\omega }_{0}\left(x\right)$ is

$${f}_{0}\left(x\right){\omega }_{0}\left(x\right)={f}_{0}\left(x\right)\frac{g\left(x\right)}{1-e\left(x\right)}\frac{1-e\left({x}^{s}\right)}{g\left({x}^{s}\right)}\propto f\left(x\right)\left\{1-e\left({x}^{s}\right)\right\}\frac{g\left(x\right)}{g\left({x}^{s}\right)}\propto \left\{f\left({x}^{h}\vert{x}^{s}\right)\frac{g\left(x\right)}{g\left({x}^{s}\right)}\right\}{f}_{0}\left({x}^{s}\right).$$

Previously, we let ${f}_{g}\left({x}^{h}\mid {x}^{s}\right)\equiv f\left({x}^{h}\vert{x}^{s}\right)g\left(x\right)/g\left({x}^{s}\right)$ define the target conditional distribution of health status. Thus, the proposed weights realize the target populations, expressed as ${f}_{g}\left({x}^{h}\mid {x}^{s}\right){f}_{1}\left({x}^{s}\right)$ and ${f}_{g}\left({x}^{h}\mid {x}^{s}\right){f}_{0}\left({x}^{s}\right)$, for minority and majority groups, respectively.

An important example arises when $g\left(x\right)=g\left({x}^{s}\right)=1$ for the ATE, where the target distribution of conditional health-status is easily obtained as $f\left({x}^{h}\vert{x}^{s}\right)$. For the ATT, $g\left(x\right)=e\left(x\right)$ and $g\left({x}^{s}\right)=e\left({x}^{s}\right)$, and therefore, the target conditional distribution of health-status is

$${f}_{g}\left({x}^{h}\mid {x}^{s}\right)=f\left({x}^{h}\vert{x}^{s}\right)\frac{e\left(x\right)}{e\left({x}^{s}\right)}=f\left(x\right)e\left(x\right){\left\{f\left({x}^{s}\right)e\left({x}^{s}\right)\right\}}^{-1}\propto {f}_{1}\left(x\right){f}_{1}{\left({x}^{s}\right)}^{-1}={f}_{1}\left({x}^{h}\vert{x}^{s}\right),$$

which is of the minority group.

We can demonstrate that the marginal distributions of health status variables are not necessarily the same between the groups in the hypothetical populations generated by the proposed weights:

$$\int \frac{{f}_{g}\left({x}^{h}\mid {x}^{s}\right){f}_{1}\left({x}^{s}\right)}{E\left[{f}_{g}\left({x}^{h}\mid {x}^{s}\right){f}_{1}\left({x}^{s}\right)\right]}d{x}^{s}\ne \int \frac{{f}_{g}\left({x}^{h}\mid {x}^{s}\right){f}_{0}\left({x}^{s}\right)}{E\left[{f}_{g}\left({x}^{h}\mid {x}^{s}\right){f}_{0}\left({x}^{s}\right)\right]}d{x}^{s}.$$

The above inequality holds unless health status and SES variables are independent or two racial groups have the same SES distribution. Therefore, we do not focus on balancing the marginal distributions of health status. Instead, we seek to balance the conditional distributions of health status given SES.

The ATO has been used as an estimand to address lack of overlap [15], which often occurs when some covariates are highly correlated with the treatment variable. To identify the ATO measure of racial disparity, we let $g\left(x\right)=e\left(x\right)\left\{1-e\left(x\right)\right\}$ and $g\left({x}^{s}\right)=e\left({x}^{s}\right)\left\{1-e\left({x}^{s}\right)\right\}$. Then, the corresponding overlap weights are ${\omega }_{1}\left(x\right)=\left\{1-e\left(x\right)\right\}/\left\{1-e\left({x}^{s}\right)\right\}$ and ${\omega }_{0}\left(x\right)=e\left(x\right)/e\left({x}^{s}\right)$. Notably, the target population for the original overlap weights is

9 $$\begin{array}{c}f\left(x\right)e\left(x\right)\left\{1-e\left(x\right)\right\}\propto {f}_{1}\left(x\right){f}_{0}\left(x\right)/f\left(x\right),\end{array}$$

which is the product of the minority and majority densities divided by the marginal density of the combined. We can show that the target conditional distribution of health status has the same form as Eq. (9):

$${f}_{g}\left({x}^{h}\mid {x}^{s}\right)=f\left({x}^{h}\vert{x}^{s}\right)\frac{e\left(x\right)\left\{1-e\left(x\right)\right\}}{e\left({x}^{s}\right)\left\{1-e\left({x}^{s}\right)\right\}}\propto \frac{{f}_{1}\left(x\right){f}_{0}\left(x\right)/f\left(x\right)}{{f}_{1}\left({x}^{s}\right){f}_{0}\left({x}^{s}\right)/f\left({x}^{s}\right)}=\frac{{f}_{1}\left({x}^{h}\vert{x}^{s}\right){f}_{0}\left({x}^{h}\vert{x}^{s}\right)}{f\left({x}^{h}\vert{x}^{s}\right)}$$

Therefore, the target conditional distribution is the overlapped conditional distribution of health status given SES between the groups.

### Estimation of health disparity measures

Based on Eqs. (4) and (5), we can construct consistent estimators for ${\mu }_{g1}$ and ${\mu }_{g0}$. Using our result that ${f}_{1}\left(x\right){\omega }_{1}\left(x\right)\propto {f}_{g}\left({x}^{h}\mid {x}^{s}\right){f}_{1}\left({x}^{s}\right)$, we can write ${\mu }_{g1}$ in Eq. (4) as

$${\mu }_{g1}=\frac{\int E\left[Y\mid Z=1,X=x\right]{f}_{1}\left(x\right){\omega }_{1}\left(x\right)dx}{\int {f}_{1}\left(x\right){\omega }_{1}\left(x\right)dx}.$$

Using our result that ${f}_{0}\left(x\right){\omega }_{0}\left(x\right)\propto {f}_{g}\left({x}^{h}\mid {x}^{s}\right){f}_{0}\left({x}^{s}\right)$, we can also write ${\mu }_{g0}$ in Eq. (5) as

$${\mu }_{g0}=\frac{\int E\left[Y\mid Z=0,X=x\right]{f}_{0}\left(x\right){\omega }_{0}\left(x\right)dx}{\int {f}_{0}\left(x\right){\omega }_{0}\left(x\right)dx}.$$

Therefore, we use the following consistent estimator for ${\tau }_{g}$:

10 $$\begin{array}{c}{\widehat{\tau }}_{g}=\frac{\sum _{i}{\omega }_{1}\left({X}_{i}\right){Z}_{i}{Y}_{i}}{\sum _{i}{\omega }_{1}\left({X}_{i}\right){Z}_{i}}-\frac{\sum _{i}{\omega }_{0}\left({X}_{i}\right){(1-Z}_{i}){Y}_{i}}{\sum _{i}{\omega }_{0}\left({X}_{i}\right)\left(1-{Z}_{i}\right)}.\end{array}$$

### Estimation of the propensity scores

In the proposed framework, the important task is to balance the conditional distributions of health status given SES between minority and majority groups. Usual methods for PS estimation may not be effective in achieving this task. However, we can view this task as estimating the PSs within each subgroup defined by SES variables, where the PSs are intended to balance health-status variables. Several researchers have investigated PS methods for subgroup analysis. Dong et al. [16] demonstrated that fitting the PS models by strata yields better covariate balance within the strata than fitting the main-effects model on the whole data. Therefore, we propose to fit the full PS model to each subgroup defined by all SES variables. As shown by Yang et al. [17], the overlap weights achieve exact balance within each subgroup when the PS models are fitted by strata and maximum likelihood (ML) is used for parameter estimation. However, exact balance is not guaranteed for the other weights, such as the IPW weights, when ML is used for parameter estimation. In their simulation study, Dong et al. [16] showed that the covariate balancing propensity score (CBPS) [18] is effective in balancing the covariates within strata when it is used to estimate the stratum-specific PS models. To have a better finite balance for the weights other than the overlap weights, we use CBPS for PS estimation.

#### Measuring concordance with IOM’s definition

In typical PS-weighted analysis, we can use the absolute standardized mean difference (ASMD) [19] to evaluate whether the covariates are balanced between groups in the target population. If ${X}_{ij}$ indicates covariate $j$ of subject $i$, then the ASMD of ${X}_{ij}$ is

$$\begin{array}{c}D\left({X}_{ij}\right)=\frac{\left|\frac{{\sum }_{i=1}^{N}{X}_{ij}{Z}_{i}{\omega }_{1}^{B}\left({X}_{i}\right)}{{\sum }_{i=1}^{N}{Z}_{i}{\omega }_{1}^{B}\left({X}_{i}\right)}-\frac{{\sum }_{i=1}^{N}{X}_{ij}{(1-Z}_{i}){\omega }_{0}^{B}\left({X}_{i}\right)}{{\sum }_{i=1}^{N}\left(1-{Z}_{i}\right){\omega }_{0}^{B}\left({X}_{i}\right)}\right|}{\sqrt{\frac{\left({s}_{1\left(j\right)}^{2}+{s}_{0\left(j\right)}^{2}\right)}{2}}},\end{array}$$

where ${s}_{1\left(j\right)}^{2}$ is the sample variance of unweighted ${X}_{ij}$ in the minority group, and ${s}_{0\left(j\right)}^{2}$ is the sample variance of unweighted ${X}_{ij}$ in the majority group.

Following IOM’s definition, we do not balance all covariates, pursued by typical causal inference approaches. Therefore, we need to modify $D\left({X}_{ij}\right)$ for health disparities research. First, we adopt the metrics introduced by Choi et al. [8] to measure the degree to which the proposed weights alter the minority SES:

11 $$\begin{array}{c}{D}_{1}\left({X}_{ij}^{s}\right)=\frac{\left|\frac{{\sum }_{i=1}^{N}{X}_{ij}^{s}{Z}_{i}}{{\sum }_{i=1}^{N}{Z}_{i}}-\frac{{\sum }_{i=1}^{N}{X}_{ij}^{s}{Z}_{i}{\omega }_{1}\left({X}_{i}\right)}{{\sum }_{i=1}^{N}{Z}_{i}{\omega }_{1}\left({X}_{i}\right)}\right|}{{s}_{1\left(j\right)}},\end{array}$$

where $j=1,\dots ,J$. The metric to measure the degree to which the proposed weights alter the majority SES is:

12 $$\begin{array}{c}{D}_{0}\left({X}_{ij}^{s}\right)=\frac{\left|\frac{{\sum }_{i=1}^{N}{X}_{ij}^{s}{(1-Z}_{i})}{{\sum }_{i=1}^{N}{(1-Z}_{i})}-\frac{{\sum }_{i=1}^{N}{X}_{ij}^{s}{(1-Z}_{i}){\omega }_{0}\left({X}_{i}\right)}{{\sum }_{i=1}^{N}{(1-Z}_{i}){\omega }_{0}\left({X}_{i}\right)}\right|}{{s}_{0\left(j\right)}},\end{array}$$

where $$j=1,\dots ,J$$.

We demonstrated that the marginal distributions of health status are generally not balanced if the weights preserve the original SES distributions. Thus, we seek to measure the extent to which health-status variables are balanced within the SES subgroups. Without loss of generality, assume that the SES variables are categorical, and the total number of subgroups that can be generated by them is $R={2}^{K}$. In our application to the RHC data, we consider four subgroups defined based on two binary SES variables, education and income. Let ${G}_{i}\in \left\{1,\dots ,R\right\}$ denote the subgroup indicator for subject $i$. Define an indicator function $1\left(A\right)$, equal to 1 if statement $A$ is true and 0 otherwise. Adopting Eq. (6) of Yang et al. [17], we modify $D\left({X}_{ij}\right)$ to measure the balance in the health-status variables in subgroup $r$:

13 $$\begin{array}{c}{D}_{r}\left({X}_{ij}^{h}\right)=\frac{\left|\frac{{\sum }_{i=1}^{N}{X}_{ij}1\left({G}_{i}=r\right){Z}_{i}{\omega }_{1}\left({X}_{i}\right)}{{\sum }_{i=1}^{N}1\left({G}_{i}=r\right){Z}_{i}{\omega }_{1}\left({X}_{i}\right)}-\frac{{\sum }_{i=1}^{N}{X}_{ij}1({G}_{i}=r){(1-Z}_{i}){\omega }_{0}\left({X}_{i}\right)}{{\sum }_{i=1}^{N}1\left({G}_{i}=r\right)\left(1-{Z}_{i}\right){\omega }_{0}\left({X}_{i}\right)}\right|}{\sqrt{\frac{\left({s}_{r1\left(j\right)}^{2}+{s}_{r0\left(j\right)}^{2}\right)}{2}}},\end{array}$$

where $j=J+1,\dots ,J+K$, $r=1,\dots ,R$, ${s}_{r1\left(j\right)}^{2}$ is the sample variance of unweighted ${X}_{ij}$ in the minority group belonging to SES subgroup $r$, and ${s}_{r0\left(j\right)}^{2}$ is the sample variance of unweighted ${X}_{ij}$ in the majority group belonging to SES subgroup $r$.

In this section, we have discussed three ASMD measures, ${D}_{1}\left({X}_{ij}^{s}\right)$, ${D}_{0}\left({X}_{ij}^{s}\right)$, and ${D}_{r}\left({X}_{ij}^{h}\right)$, to evaluate whether given weights create target populations concordant with IOM’s definition. Smaller values of these three indicate that the given weights are reliable. Austin and Stuart [19] recommended using 0.1 as a threshold to detect covariate imbalance. Following this recommendation, if there are any covariates whose ASMD values are greater than 0.1, this will indicate that the weights may not achieve the IOM’s definition successfully.

### Characterizing target populations

It would be important to characterize the target populations identified by the proposed weights because they are usually hypothetical and not directly observable from the data. Adopting the estimators for ${\mu }_{g1}$ and ${\mu }_{g0}$ in (10), we can estimate the means of ${X}_{ij}$ in the target joint distributions. The estimator for the mean of ${X}_{ij}$ the weighted minority group is

14 $$\begin{array}{c}{m}_{1}\left({X}_{ij}\right)=\frac{\sum _{i}{\omega }_{1}\left({X}_{i}\right){Z}_{i}{X}_{ij}}{\sum _{i}{\omega }_{1}\left({X}_{i}\right){Z}_{i}},\end{array}$$

and that for the weighted majority group is

15 $$\begin{array}{c}{m}_{0}\left({X}_{ij}\right)=\frac{\sum _{i}{\omega }_{0}\left({X}_{i}\right){(1-Z}_{i}){X}_{ij}}{\sum _{i}{\omega }_{0}\left({X}_{i}\right){(1-Z}_{i})}.\end{array}$$

The estimators ${m}_{1}\left({X}_{ij}\right)$ and ${m}_{0}\left({X}_{ij}\right)$ are obtained by replacing outcome ${Y}_{i}$ with covariate ${X}_{ij}$ in those of ${\mu }_{g1}$ and ${\mu }_{g0}$.

### Application to right heart catheterization data

Connors et al. [10] examined the effectiveness of RHC on critically ill patients during the first 24 h after admission to the intensive care unit. The authors compared survival outcomes and found that patients managed with RHC had poorer survival outcomes than those without RHC. The original study [10] and other studies [9, 20, 21] focused on whether RHC was beneficial or harmful for critically ill patients. In this study, we examined whether there was a disparity in receiving RHC between black and white patients admitted to the intensive care unit. If there is a difference in the probabilities of receiving RHC even after adjusting for the variables relating to clinical needs and health status, while keeping the original SES distributions, that difference would indicate some racial disparities in receiving RHC. Because physicians believed that management with RHC leads to better patient outcomes at the time of the RHC study [10], even though the actual RHC study demonstrated that it was not beneficial in general, reduced use of RHC by black patients would reflect disparities. We compared the unadjusted proportions of patients receiving RHC, which indicated that black patients were 1.86% less likely to receive RHC than white patients. This proportion difference was not statistically significant, with a p-value of 0.286 at a 5% level. However, this estimate did not account for the differences in clinical needs and health status, which should not contribute to health disparity measures.

The original data includes 920 blacks, 4460 whites, and 335 others, but we considered only black and white patients to illustrate our methods. Thus, our data consisted of 5380 patients. We considered twenty continuous (age, number of comorbid illnesses, and baseline laboratory values) and four categorical (sex, primary disease category, cancer, and ‘do not resuscitate’ (DNR) status) variables for health status. After generating the dummy variables for the categorical variables, we obtained twenty-seven health-status variables. Table S1 provides a full list of the health-status variables. For SES, we considered years of education and income. We converted years of education to a binary variable, equal to 1 if high school education or more and 0 otherwise. Income had four categories, but these categories were pooled to have the following two categories: ‘less than $25K’ and ‘$25K or greater’. Table 1 presents the baseline characteristics of non-RHC and RHC patients by race. Table 2 shows the frequency distribution of the SES subgroups.

**Table 2:** Joint distribution of education and income in right heart catheterization data

Education	Income	
	Less than $25K	$25K or greater
Less than high school	3321	532
High school or more	801	726

In the context of this analysis, the PS was defined as the probability of being a black patient conditional on the covariates. The goal of this analysis was to demonstrate the use of the deweighting approach to estimate the ATE, ATT, and ATO measures of health disparities in receiving RHC. The full PS model for the deweighting approach included the main effects of all health-status variables but was estimated within each of the SES subgroups, using the CBPS. Thus, the full PSs were obtained from these SES Stratum-specific Health-Status (SSHS)-PS models. The SES-PS model was fitted with the main effects of education and income, and their interaction term. Because the SES variables were categorical, the model was saturated. Finally, the proposed weights for the ATE, ATT, and ATO were calculated using the estimated full PSs and SES-PSs.

Based on Eq. (10), the proposed weights were used to estimate the ATE, ATT, and ATO measures of racial disparities. We used the survey R package [22] to calculate 95% confidence intervals and presented inference results in Table 3. We used four methods to estimate each disparity measure: (i) full PS using the main effects of health-status and SES variables, (ii) full PS based on SSHS-PS, (iii) health-status PS, and (iv) deweighting. These full or health-status PSs were estimated using the CBPS. Note that methods (i) and (ii) used the PSs adjusting for all covariates, and methods (iii) and (iv) adjusted for only the health-status variables. Even though all estimates were not statistically significant at a 5% level, we observed meaningful differences between the methods regarding point estimates. The ATT estimates based on the full PS and SSHS-PS were positive, indicating that black patients were more likely to receive RHC by 1.51% and 0.64%, respectively. Therefore, using all covariates over-adjusted the crude difference, and as a result, the sign of the estimate was reversed. The ATT estimates based on the health-status PS and deweighting reduced the disparities to -0.14% and − 0.35%, respectively, from − 1.86%. Similar observations were found for the ATO estimates. For the ATE, only the deweighting estimate (-2.44%) represented a larger disparity than the unadjusted estimate (-1.86%).

**Table 3:** Racial disparity estimates (%), as measured by the average controlled differences, in receipt of a right heart catheterization

Estimand	Method	Point estimate	Lower bound	Upper bound
NA	Unadjusted difference	-1.86	-5.25	1.59
ATE	Full PS	-0.16	-4.70	4.38
	SES stratum-specific health-status PS	-1.33	-6.27	3.62
	Health-status PS	-0.03	-3.75	3.69
	Deweighting	-2.44	-6.65	1.77
ATT	Full PS	1.51	-2.28	5.30
	SES stratum-specific health-status PS	0.64	-3.13	4.41
	Health-status PS	-0.14	-3.76	3.47
	Deweighting	-0.35	-4.12	3.41
ATO	Full PS	0.98	-2.69	4.64
	SES stratum-specific health-status PS	0.27	-3.38	3.92
	Health-status PS	-0.48	-4.05	3.08
	Deweighting	-0.88	-4.54	2.77

*NA *Not available, *ATE *Average treatment effect, *ATT *Average treatment effect in the treated, *ATO *Average treatment effect in the overlap population, *PS *Propensity score, *SES *Socioeconomic status

We used our three metrics in Eqs. (11, 12, 13) to examine how better the proposed weights achieved the IOM’s definition than the balancing weights based on the SSHS-PS, which we will call SSHS weights. Note that the proposed weights are obtained as the SSHS weights divided by the balancing weights based on the SES-PS. First, we checked whether the marginal SES distributions of blacks and whites were preserved in the ATE weighted data. To this end, we looked at whether the values of ${D}_{0}\left({X}_{ij}^{s}\right)$ were sufficiently small for education and income. The values of ${D}_{0}\left({X}_{ij}^{s}\right)$ for education and income were (0.020, 0.050) when the proposed weights were used and (0.024, 0.008) when the SSHS weights were used. The values of ${D}_{1}\left({X}_{ij}^{s}\right)$ for education and income were (0.019, 0.035) when the proposed weights were used and (0.275, 0.426) when the SSHS weights were used. Therefore, the black distribution of SES was significantly changed by the SSHS weights. Next, we investigated whether the values of ${D}_{0}\left({X}_{ij}^{s}\right)$ were sufficiently small for education and income in the ATT weighted data. The values of ${D}_{0}\left({X}_{ij}^{s}\right)$ were approximately 0.0003 for education and income when the proposed weights were used and (0.257, 0.324) with the SSHS weights. Thus, the white distribution of SES was significantly changed by the SSHS weights. All values of ${D}_{1}\left({X}_{ij}^{s}\right)$ were 0 because a unit weight was applied to all black patients. Finally, we checked both ${D}_{1}\left({X}_{ij}^{s}\right)$ and ${D}_{0}\left({X}_{ij}^{s}\right)$ in the ATO population. The values of ${D}_{0}\left({X}_{ij}^{s}\right)$ were (0.004, 0.0001) with the proposed weights and (0.229, 0.290) with the SSHS weights. Those of ${D}_{1}\left({X}_{ij}^{s}\right)$ were (0.008, 0.002) with the proposed weights and (0.088, 0.122) with the SSHS weights. These numerical results demonstrated that the proposed weights sufficiently preserved the original SES distributions for both groups in the ATE, ATT, and ATO populations, while the SSHS weights failed to do so.

**Fig. 1:**
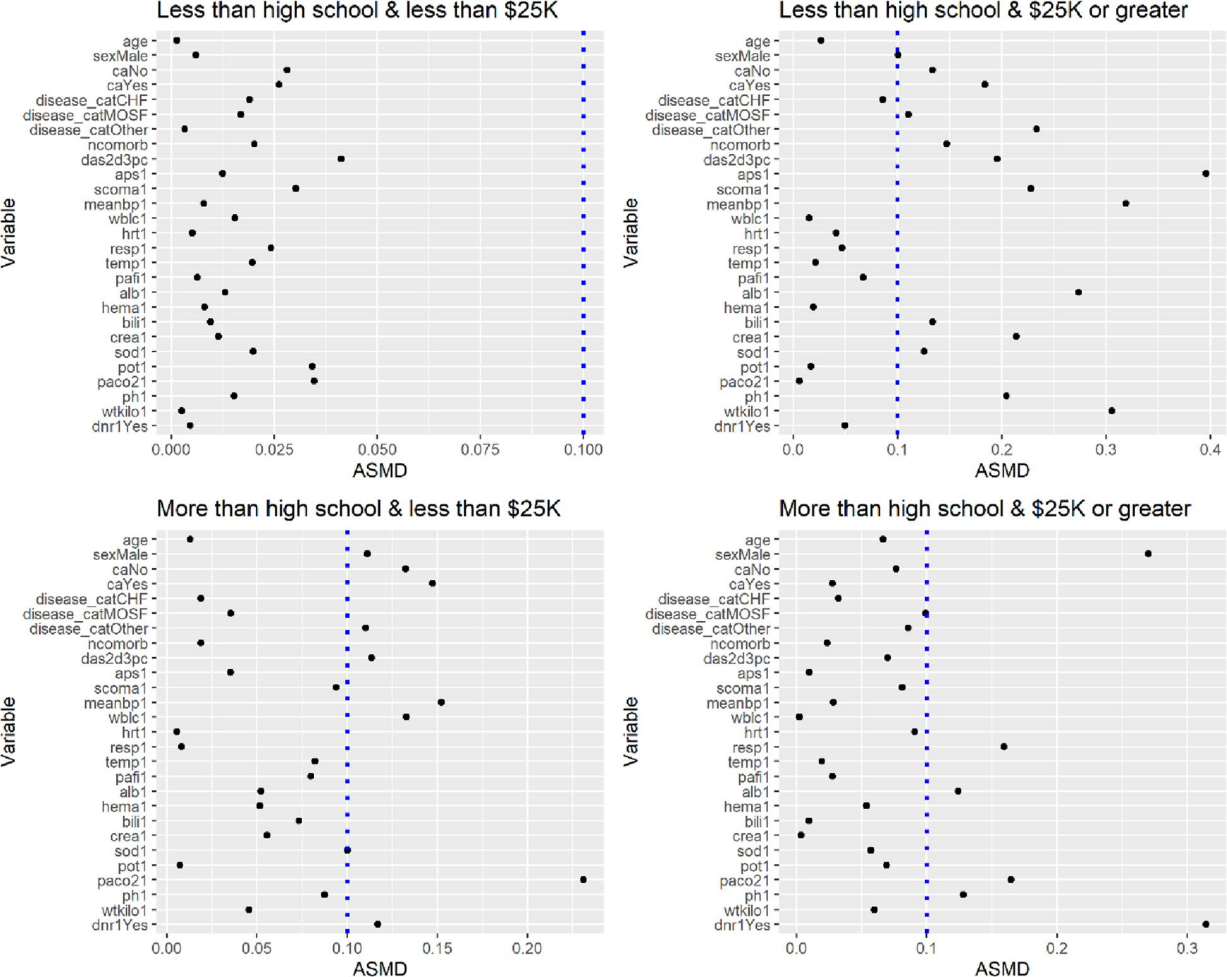
Absolute standardized mean differences (Eq. 13) of the health-status variables in the combined group within each of the four subgroups defined by education and income in right heart catheterization data. The full propensity scores of black patients were estimated by maximum likelihood

**Fig. 2:**
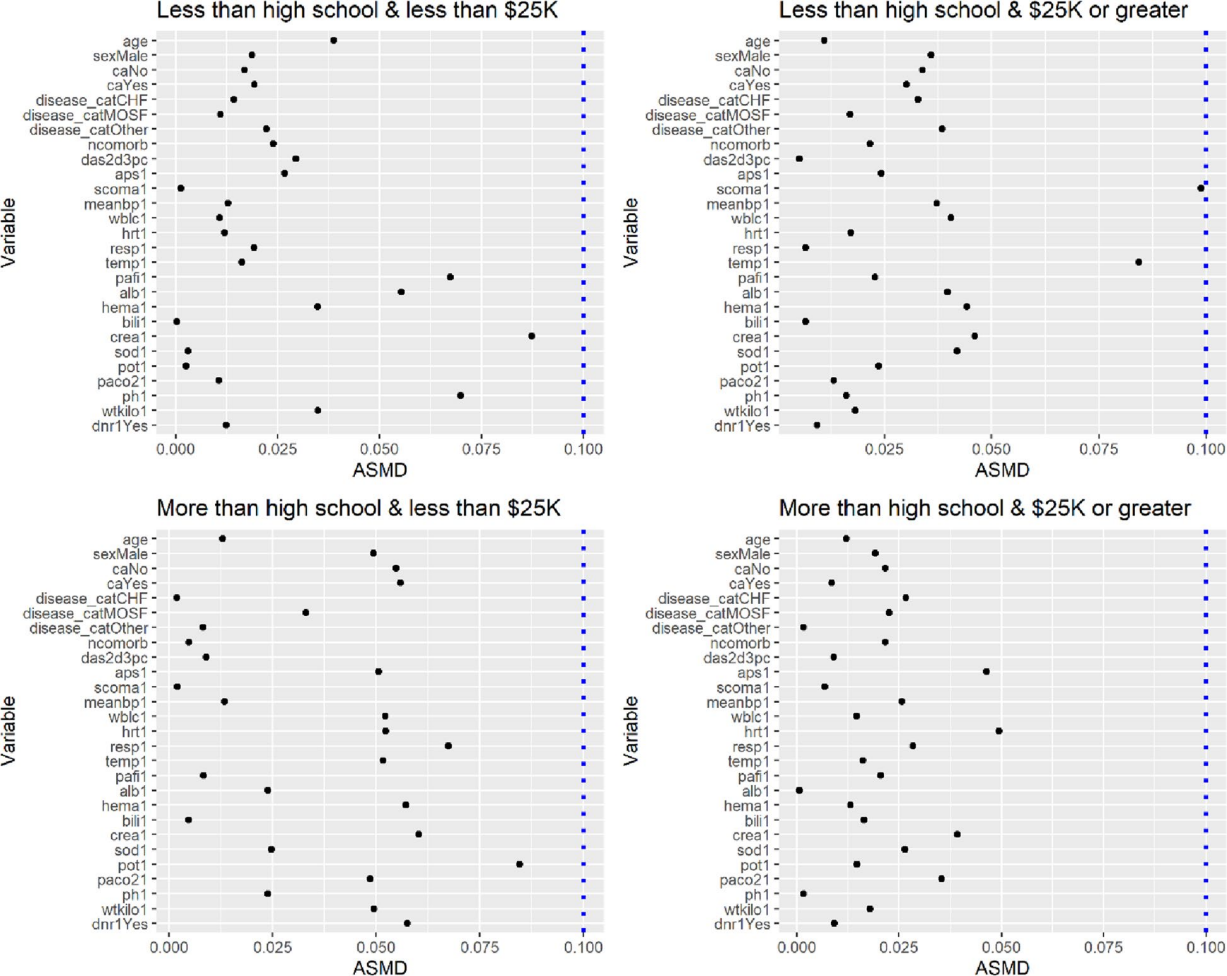
Absolute standardized mean differences (Eq. 13) of the health-status variables in the black group within each of the four subgroups defined by education and income in right heart catheterization data. The full propensity scores of black patients were estimated by maximum likelihood

In addition to the degree to which SES variables were altered, we checked the covariate balance of the health-status variables after the proposed weights were applied. Particularly, we investigated how CBPS and ML performed differently in terms of balancing health status variables when they were used to estimate the SSHS weights. In the ATE population, within each SES subgroup, we checked whether the health-status variables of blacks and whites were balanced. When ML was used, there was a severe imbalance in the health-status variables between groups: except for the SES subgroup that was educated less than high school and had an income of less than $25K, many health-status variables had the values of $${D}_{r}\left({X}_{ij}^{h}\right)$$ over 0.1 (Fig. 1). However, when CBPS was used, all values of $${D}_{r}\left({X}_{ij}^{h}\right)$$ across the SES subgroups were smaller than 0.1. In the ATT population, within each of the SES subgroups, we checked whether the health-status variables of whites became similar to those of blacks after weighting. When ML was used, all values of $${D}_{r}\left({X}_{ij}^{h}\right)$$ for the health-status variables within each SES subgroup were smaller than 0.1, but a few values were near 0.1 (Fig. 2). For example, the values of $${D}_{r}\left({X}_{ij}^{h}\right)$$ for Glasgow Coma Score and temperature were 0.099 and 0.084, respectively, in the subgroup that was educated less than high school but had an income of $25K or greater. However, when CBPS was used, the covariate balance of all health-status variables in the ATT population was satisfactory with all values of $${D}_{r}\left({X}_{ij}^{h}\right)$$ almost 0. Those of all health-status variables in the ATO population were also almost 0.

We used the estimators $${m}_{1}\left({X}_{ij}\right)$$ and $${m}_{0}\left({X}_{ij}\right)$$ in Eqs. (14) and (15) to characterize black and white patients in the ATE, ATT, and ATO populations. Table 4 shows the characteristics of the ATE-weighted black and white patients, and the combined sample. The marginal means of the health-status variables between the weighted black and white patients were similar to those of the combined sample. The education and income of the weighted white patients were minimally altered. In the original samples, 18.6% of black patients had a high-school education or more, but 30.4% of white patients were so educated. After the proposed weighting, those percentages were changed to 19.3% and 31.3%, respectively. Regarding income, 11.6% of black patients earned $25K or greater, while 25.8% of white patients earned so. After the proposed weighting, those percentages were changed to 12.8% and 28.0%, respectively. Table 5 shows the original covariates of black patients, and the ATT-weighted and original covariates of white patients. The marginal means of the health-status variables between the black and weighted white patients were similar. The education and income of the weighted white patients were completely unaltered. As a result, the original differences in these SES variables were preserved. Table 6 shows the ATO-weighted covariates in black and white patients. Again, we observed that education and income were not altered, and the marginal distributions of the health-status variables were similar between the weighted groups.

**Table 4:** Characteristics of the ATE-weighted black and white patients, and the original combined sample in right heart catheterization data

	Weighted black patients	Weighted white patients	Overall
N	940.74	4652.67	5380
Age (mean (SD))	62.28 (16.75)	61.04 (16.79)	61.99 (16.46)
Sex = Male (%)	511.1 (54.3)	2552.5 (54.9)	2994 (55.7)
Cancer (%)			
Metastatic	62.2 (6.6)	353.7 (7.6)	362 (6.7)
No cancer	721.8 (76.7)	3506.4 (75.4)	4105 (76.3)
Localized	156.7 (16.7)	792.6 (17.0)	913 (17.0)
Disease category (%)			
ARF	410.5 (43.6)	2029.8 (43.6)	2354 (43.8)
CHF	71.7 (7.6)	353.5 (7.6)	422 (7.8)
MOSF	260.2 (27.7)	1343.8 (28.9)	1504 (28.0)
Other	198.4 (21.1)	925.6 (19.9)	1100 (20.4)
Number of comorbidities (mean (SD))	1.53 (1.16)	1.49 (1.15)	1.51 (1.16)
Duke activity status index (mean (SD))	20.23 (5.36)	20.59 (5.36)	20.48 (5.31)
APACHE score (mean (SD))	54.98 (19.91)	55.10 (20.06)	54.59 (19.88)
Glasgow coma score (mean (SD))	21.78 (29.75)	21.76 (31.25)	21.03 (30.48)
Mean blood pressure (mean (SD))	79.26 (40.22)	78.91 (38.59)	78.56 (38.16)
WBC (mean (SD))	15.85 (13.10)	15.64 (11.65)	15.77 (11.98)
Heart rate (mean (SD))	114.17 (43.36)	114.92 (41.87)	114.98 (41.52)
Respiratory rate (mean (SD))	27.96 (14.94)	27.93 (14.06)	28.05 (14.12)
Temperature (mean (SD))	37.59 (1.79)	37.65 (1.78)	37.61 (1.78)
PaO2/FiO2 ratio (mean (SD))	223.90 (111.31)	223.53 (120.81)	221.31 (114.15)
Albumin (mean (SD))	3.08 (0.65)	3.07 (0.81)	3.09 (0.79)
Hematocrit (mean (SD))	32.01 (8.43)	31.76 (8.38)	31.95 (8.38)
Bilirubin (mean (SD))	2.05 (4.18)	2.13 (4.41)	2.11 (4.37)
Creatinine (mean (SD))	2.20 (2.06)	2.19 (2.18)	2.13 (2.05)
Sodium (mean (SD))	136.91 (7.52)	136.83 (7.67)	136.81 (7.63)
Potassium (mean (SD))	4.08 (1.02)	4.07 (1.03)	4.07 (1.02)
PaCO2 (mean (SD))	39.11 (13.28)	38.62 (13.29)	38.80 (13.15)
PH (mean (SD))	7.38 (0.10)	7.39 (0.11)	7.39 (0.11)
Weight (mean (SD))	68.45 (29.27)	68.36 (29.22)	68.25 (29.05)
‘Do not resuscitate’ status on day 1 = Yes (%)	112.1 (11.9)	508.4 (10.9)	626 (11.6)
High school education or more (%)	182.0 (19.3)	1457.2 (31.3)	1527 (28.4)
Income >=$25k (%)	120.1 (12.8)	1301.7 (28.0)	1258 (23.4)

**Table 5:** Characteristics of black, the ATT-weighted, and original white patients in right heart catheterization data

	Black patients	Weighted white patients	White patients
N	920	4461.97	4460
Age (mean (SD))	56.50 (17.50)	55.01 (17.78)	63.13 (16.00)
Sex = Male (%)	455.0 (49.5)	2215.0 (49.6)	2539 (56.9)
Cancer (%)			
Metastatic	67.0 (7.3)	357.3 (8.0)	295 (6.6)
No cancer	742.0 (80.7)	3563.5 (79.9)	3363 (75.4)
Localized	111.0 (12.1)	541.2 (12.1)	802 (18.0)
Disease category (%)			
ARF	376.0 (40.9)	1865.8 (41.8)	1978 (44.3)
CHF	79.0 (8.6)	389.9 (8.7)	343 (7.7)
MOSF	286.0 (31.1)	1412.9 (31.7)	1218 (27.3)
Other	179.0 (19.5)	793.4 (17.8)	921 (20.7)
Number of comorbidities (mean (SD))	1.39 (1.13)	1.34 (1.11)	1.54 (1.16)
Duke activity status index (mean (SD))	20.58 (5.61)	20.97 (5.58)	20.46 (5.25)
APACHE score (mean (SD))	56.30 (20.92)	56.12 (20.60)	54.24 (19.64)
Glasgow coma score (mean (SD))	24.45 (31.87)	23.72 (32.46)	20.33 (30.14)
Mean blood pressure (mean (SD))	85.34 (42.59)	85.71 (40.84)	77.16 (37.04)
WBC (mean (SD))	16.12 (13.66)	16.21 (12.34)	15.70 (11.60)
Heart rate (mean (SD))	115.61 (43.64)	117.06 (41.78)	114.85 (41.08)
Respiratory rate (mean (SD))	28.68 (15.28)	28.83 (14.16)	27.93 (13.87)
Temperature (mean (SD))	37.46 (1.95)	37.54 (1.92)	37.65 (1.74)
PaO2/FiO2 ratio (mean (SD))	244.08 (122.02)	241.38 (130.85)	216.62 (111.90)
Albumin (mean (SD))	3.04 (0.69)	3.04 (0.92)	3.10 (0.81)
Hematocrit (mean (SD))	30.50 (8.39)	30.52 (8.31)	32.25 (8.34)
Bilirubin (mean (SD))	2.12 (4.33)	2.12 (4.10)	2.11 (4.37)
Creatinine (mean (SD))	2.63 (2.71)	2.54 (2.76)	2.03 (1.87)
Sodium (mean (SD))	137.56 (7.74)	137.35 (8.00)	136.66 (7.59)
Potassium (mean (SD))	4.12 (1.12)	4.14 (1.11)	4.06 (1.00)
PaCO2 (mean (SD))	37.74 (12.46)	37.33 (12.71)	39.02 (13.28)
PH (mean (SD))	7.38 (0.11)	7.39 (0.11)	7.39 (0.11)
Weight (mean (SD))	68.92 (29.87)	69.18 (29.98)	68.11 (28.88)
‘Do not resuscitate’ status on day 1 = Yes (%)	79.0 (8.6)	347.9 (7.8)	547 (12.3)
High school education or more (%)	171.0 (18.6)	1356.0 (30.4)	1356 (30.4)
Income >=$25k (%)	107.0 (11.6)	1151.0 (25.8)	1151 (25.8)

**Table 6:** Characteristics of the ATO-weighted black and white patients in right heart catheterization data

	Weighted black patients	Weighted white patients
N	823.21	3979.7
Age (mean (SD))	58.38 (17.16)	56.80 (17.36)
Sex = Male (%)	417.4 (50.7)	2019.3 (50.7)
Cancer (%)		
Metastatic	59.1 (7.2)	312.3 (7.8)
No cancer	652.3 (79.2)	3127.5 (78.6)
Localized	111.8 (13.6)	539.9 (13.6)
Disease category (%)		
ARF	346.0 (42.0)	1700.0 (42.7)
CHF	70.7 (8.6)	346.6 (8.7)
MOSF	242.9 (29.5)	1205.9 (30.3)
Other	163.6 (19.9)	727.2 (18.3)
Number of comorbidities (mean (SD))	1.43 (1.14)	1.38 (1.12)
Duke activity status index (mean (SD))	20.44 (5.57)	20.83 (5.47)
APACHE score (mean (SD))	55.47 (20.55)	55.43 (20.53)
Glasgow coma score (mean (SD))	23.40 (30.99)	22.99 (31.91)
Mean blood pressure (mean (SD))	83.39 (41.54)	83.68 (40.02)
WBC (mean (SD))	15.94 (13.30)	15.88 (11.89)
Heart rate (mean (SD))	115.38 (43.53)	116.74 (41.58)
Respiratory rate (mean (SD))	28.47 (15.22)	28.64 (14.14)
Temperature (mean (SD))	37.52 (1.89)	37.59 (1.85)
PaO2/FiO2 ratio (mean (SD))	235.88 (117.25)	234.07 (122.65)
Albumin (mean (SD))	3.05 (0.68)	3.05 (0.88)
Hematocrit (mean (SD))	31.08 (8.40)	31.01 (8.25)
Bilirubin (mean (SD))	2.09 (4.30)	2.12 (4.15)
Creatinine (mean (SD))	2.37 (2.30)	2.33 (2.40)
Sodium (mean (SD))	137.31 (7.57)	137.13 (7.85)
Potassium (mean (SD))	4.08 (1.06)	4.09 (1.07)
PaCO2 (mean (SD))	38.35 (12.78)	37.86 (13.02)
PH (mean (SD))	7.38 (0.11)	7.39 (0.11)
Weight (mean (SD))	68.70 (29.94)	68.89 (29.82)
‘Do not resuscitate’ status on day 1 = Yes (%)	74.9 (9.1)	322.3 (8.1)
High school education or more (%)	150.3 (18.3)	1201.9 (30.2)
Income >=$25k (%)	95.7 (11.6)	1029.1 (25.9)

## Discussion

In our empirical example, to balance health status conditional on SES, we fitted the full PS model to each of the four strata based on education and income. However, if the number of SES subgroups is large, then fitting the full PS models by the SES strata can produce unstable PS estimates, which results in poor balance of health status. This problem can occur if some strata sizes are too small, or extreme PSs exist. In such a case, more sophisticated methods than the subgroup fits would be needed. For example, Dong et al. [16] devised a stochastic search algorithm that selects one of two competing models for each subgroup, where model comparisons are performed based on covariate balance. Yang et al. [17] demonstrated that LASSO [23] is effective in reducing the number of interactions between the covariates to be balanced and strata variables for subgroup analysis of ATO. Validating the use of these methods for the deweighting approach merits further investigation.

As in traditional associational or causal analyses, the interpretation and validity of our approach depend on what data are measured. Both the health-status PS and deweighting approaches commonly adjust for health-status variables, and thus, the resultant estimates represent racial differences when the observed health status or clinical needs are balanced toward a specific target population. However, these approaches are different in terms of how unmeasured SES variables affect their interpretations and validities. In the health-status PS approach, the PS model is fitted using only the health-status variables, and SES variables are not directly involved in the estimation of the PSs. This approach theoretically captures all measured and unmeasured SES variables in the analysis. However, it is impossible to assess how the unobserved SES variables are affected by weighting. In the deweighting approach, the SES variables are directly involved in preserving the SES distributions in comparison groups. Therefore, it can efficiently protect the observed SES distributions from being altered by weighting. However, this theoretically focuses only on the measured SES variables, which excludes the existence of potential unmeasured SES variables.

It must be beneficial to investigate other relevant definitions of health disparities. For example, the World Health Organization (WHO) defines health inequities as “unfair, avoidable, or systematic differences in the health status and access to health resources of different population groups, whether those groups are defined socially, economically, demographically, or geographically or by other dimensions of inequality (e.g., sex, gender, ethnicity, disability, or sexual orientation)” [24]. The WHO also states that “health and health inequity are determined by the conditions in which people are born, grow, live, work, play and age, as well as biological determinants” [24]. Therefore, the WHO definition includes a broader range of SES factors as determinants of health inequities. The proposed approach could implement the WHO definition by using more extensive SES measurements, including sex, elderly, housing, occupation, and disability, to generate the SES subgroups and SES-PS. Numerical evaluations of the deweighting approach using racial disparity data with comprehensive SES measurements merit future investigation.

## Conclusion

In this study, we proposed a novel PS approach for estimating health disparities concordant with IOM’s definition. The existing PS methods suffer from the critical limitation that SES variables cannot be preserved reliably. The approach overcomes this limitation by deweighting the subjects by SES-based PSs. We formally defined the estimands of health disparities and presented consistent estimators based on the proposed weights. Our analysis of RHC data demonstrated that the proposed deweighting is an effective method to estimate the measures of health care disparities.

### Supplementary Information

**Supplementary Material 1.**

## Data Availability

The dataset used during the current study is available from https://hbiostat.org/data/.

## References

[1] Jackson JW, VanderWeele TJ (2018). Decomposition analysis to identify intervention targets for reducing disparities. Epidemiology.

[2] Ben-Michael E, Feller A, Kelz R, Keele L. Estimating Racial Disparities in Emergency General Surgery. 2023. arXiv preprint arXiv:2209.04321.

[3] Unequal Treatment (2003). Confronting racial and ethnic disparities in health care (with CD).

[4] Duan N, Meng X, Lin JY, Chen C, Alegria M (2008). Disparities in defining disparities: statistical conceptual frameworks. Stat Med.

[5] Cook BL, McGuire TG, Meara E, Zaslavsky AM (2009). Adjusting for health status in non-linear models of health care disparities. Health Serv Outcomes Res Methodol.

[6] McGuire TG, Alegria M, Cook BL, Wells KB, Zaslavsky AM (2006). Implementing the Institute of Medicine definition of disparities: an application to mental health care. Health Serv Res.

[7] Li F, Li F (2019). Propensity score weighting for causal inference with multiple treatments. Ann Appl Stat.

[8] Choi BY, Gelfond J, Kaushik D, Svatek RS, Wang C-P (2023). Health status balancing weights for estimation of health care disparities. Health Serv Outcomes Res Method.

[9] Li F, Morgan KL, Zaslavsky AM (2018). Balancing covariates via propensity score weighting. J Am Stat Assoc.

[10] Connors AF (1996). The effectiveness of right heart catheterization in the initial care of critically ill patients. SUPPORT Investigators. JAMA.

[11] Li F, Thomas LE, Li F (2018). Addressing extreme propensity scores via the overlap weights. Am J Epidemiol.

[12] Li F, Zaslavsky AM, Landrum MB (2013). Propensity score weighting with multilevel data. Stat Med.

[13] Hirano K, Imbens GW, Ridder G (2003). Efficient estimation of average treatment effects using the estimated propensity score. Econometrica.

[14] Rosenbaum PR, Rubin DB (1983). The central role of the propensity score in observational studies for causal effects. Biometrika.

[15] Crump RK, Hotz VJ, Imbens GW, Mitnik OA (2009). Dealing with limited overlap in estimation of average treatment effects. Biometrika.

[16] Dong J, Zhang JL, Zeng S, Li F (2020). Subgroup balancing propensity score. Stat Methods Med Res.

[17] Yang S, Lorenzi E, Papadogeorgou G, Wojdyla DM, Li F, Thomas LE (2021). Propensity score weighting for causal subgroup analysis. Stat Med.

[18] Imai K, Ratkovic M (2014). Covariate balancing propensity score. J R Stat Soc B.

[19] Austin PC, Stuart EA (2015). Moving towards best practice when using inverse probability of treatment weighting (IPTW) using the propensity score to estimate causal treatment effects in observational studies. Statist Med.

[20] Hirano K, Imbens GW (2001). Estimation of Causal effects using propensity score weighting: an application to data on right heart catheterization. Health Serv Outcomes Res Method.

[21] Choi BY (2021). Subclassification estimation of the weighted average treatment effect. Biom J.

[22] Lumley T (2004). Analysis of complex survey samples. J Stat Soft.

[23] Tibshirani R (1996). Regression shrinkage and selection via the lasso. J Roy Stat Soc Ser B (Methodol).

[24] World Health Organization. Health equity. 2024. https://www.who.int/health-topics/health-equity.

