# Supplementary material for: Propensity score analysis for health care disparities: a deweighting approach
Source: BMC Med Res Methodol. 2024 May 3;24:106. doi: 10.1186/s12874-024-02230-5 (PMC11067258; doi:10.1186/s12874-024-02230-5)
Supplement: Supplementary file 1 — Supplementary Material 1. [file 12874_2024_2230_MOESM1_ESM.pdf]

Propensity score analysis for health care disparities: a deweighting approach

Byeong Yeob Choi[1]

[1]Department of Population Health Sciences, UT Health San Antonio, San Antonio, TX, USA

Supplementary Table

Variable	Description
age	Age (years)
sex	Male
caNo	No Cancer
caYes	Localized Cancer
disease_catCHF	Primiary disease category - CHF
disease_catMOSF	Primiary disease category - MOSF
disease_catOther	Primiary disease category - Other
ncomorb	Number of comorbidities
das2d3pc	DASI - Duke Activity Status Index
aps1	APACHE score
scoma1	Glasgow coma score
meanbp1	Mean Blood Pressure
wblc1	WBC
hrt1	Heart Rate
resp1	Respiratory Rate
temp1	Temparature
pafi1	PaO2/FI02 ratio
alb1	Albumin
hema1	Hematocrit
bili1	Bilirubin
crea1	Creatinine
sod1	Sodium
pot1	Potassium
paco21	PaCO2
ph1	PH
wtkilo1	Weight
dnr1	Do Not Resuscitate status on day 1
edu	High school education or more
income	$25K or greater

Supplementary Table S1: Abbreviations of the variables considered in the right heart catheterization example.
